# Effects of perceived stress on turnover intention of female healthcare staff: a serial multiple mediation model

**DOI:** 10.1186/s12889-024-18654-z

**Published:** 2024-04-29

**Authors:** Dongling Yuan, Muli Hu, Na Yao, Huiyuan Zhong, Yinghong Xiao, Xiao Zhou, Ruoyi Zhang, Yi Zhang

**Affiliations:** 1 Medical Psychological Center, the Second Xiangya Hospital, Central South University, 139 Renmin Rd, Changsha, 410011 Hunan China; 2 Department of Human Resources, the Second Xiangya Hospital, Central South University, Changsha, 410011 China; 3 Medical Psychological Institute, Central South University, Changsha, 410011 China; 4 National Clinical Research Center On Mental Disorders (Xiangya), Changsha, 410011 China; 5 Department of Human Resources & Office of Talents Recruitment and Development of Central, South University, Changsha, 410011 China; 6 Central South University Education Foundation, Changsha, 410011 China

**Keywords:** Perceived stress, Turnover intention, Female healthcare staff, Preventive coping, Proactive coping, Work-family balance, Serial multiple mediation model

## Abstract

**Background:**

Healthcare staff in China, especially females, work in a high-pressure, high-load, and high-risk environment, which affects the physical and mental health, the efficiency and quality of work, and increases turnover intention. The present study investigated the relationship between perceived stress and turnover intention in female healthcare staff, and the effects of future-oriented coping and work-family balance on this relationship.

**Methods:**

Four hundred thirty-five female medical workers were recruited to perform a perceived stress scale, future-oriented coping inventory, work-family balance scale and turnover intention scale. Meanwhile, serial multiple mediation analysis was performed using PROCESS.

**Results:**

1) Perceived stress positively predicted the level of turnover intention in female healthcare staff; 2) Preventive coping and proactive coping showed mediation effects on the relationship between perceived stress and turnover intention, and preventive coping positively related to proactive coping; 3) The work-family balance also showed mediation effects on the relationship between perceived stress and turnover intention; 4) Preventive coping, proactive coping and work-family balance showed a serial multiple mediation on the relationship between perceived stress and turnover intention in female healthcare workers.

**Conclusions:**

Perceived stress affects the level of turnover intention in female healthcare staff through preventive coping, proactive coping, and work-family balance. In addition, the sequential model of future-oriented coping was validated among female healthcare staff.

## Background

In recent years, the turnover of healthcare staff has increased dramatically in China, which has become an important issue affecting the sustainable and healthy development of the medical system. A recent survey shows that 55.6% of healthcare staff have moderate or above turnover intentions [1]. Meanwhile, the turnover rate of female healthcare staff is about 20% to 45% [2, 3]. Turnover intention is commonly thought an important antecedent variable of turnover and positively related to actual turnover [4]. Furthermore, a follow-up study by Huang et al. found that the turnover intention of healthcare workers was negatively related to the quality of healthcare [5]. Therefore, it is significant to explore the factors that affect female healthcare staff's turnover intention to reduce their turnover rate and increase the quality of healthcare.

High stress is one of the important causes of turnover intention in the healthcare staff, such as overload [6], repellent communication [7], the risk of exposure to infectious diseases [8], work-family conflict and role ambiguity [9]. The survey has shown that 2.2% to 14.5% of female healthcare staff feel great pressure [10]. However, Chinese healthcare workers, especially females, may experience more and different stressors. For example, Chinese traditional culture considers that " A woman's duty is to do homework and bear babies to carry on the family line " and "women are better suited than men to take care of children and families". Even modern professional women still do more housework and child care than husbands. These further increase stress levels among female healthcare workers in China. Meanwhile, stress is prone to cause female healthcare workers subjective psychological fluctuations and shows a series of psychological and behavioral responses, such as an increased turnover intention. A previous study has demonstrated that family demands (such as taking care of the elderly, raising and educating children) have a significantly stronger negative impact on the work of female healthcare workers than male [11]. Overall, female healthcare workers in China may experience more and specific stressors. It is important to study the relationship between perceived stress and turnover intention in female healthcare workers.

At the same time, Lazarus' transactional model of stress and coping [12] pointed out that stress is a process changing with time and the environment, and stress coping plays a key role in this process. The mode proposes that stress coping refers to the cognitive and behavioral efforts that an individual exerts to manage the internal and external demands of human–environment interactions, which are evaluated as taxing or exceeding the individual's resources. Simultaneously, individuals will show different coping strategies in the case of acute and chronic stress. Specifically, individuals will produce a series of physiological and psychological behavioral responses to cope with acute stress events, called reactive coping [13]. In terms of long-term chronic stress, except for some maladaptive individuals, most individuals can successfully cope with stress through resource accumulation, long-term planning, and skill improvement. This coping strategy is called future-oriented coping, referring to a process that individual's pre-action on expected or potential stressors to prevent its occurrence or its adverse effects. Schwarzer et al. [14] further differentiated future-oriented coping into proactive coping and preventive coping. Specifically, proactive coping refers to self-improvement and growth by focusing on challenging stressors (such as job-hopping); preventive coping refers to the accumulation of resources in advance in response to potentially threatening stressors (such as unemployment) [15]. Gan et al. [16] found that both proactive and preventive coping are positively associated with positive affection and negatively associated with perceived stress, suggesting that both coping styles not only promote positive outcomes but also mitigate negative ones. Hu et al. [17] also found proactive coping is beneficial to promote healthcare staff to seek positive meanings from high-intensity stress work, and relieve individual stress levels and job burnout. However, there is a lack of reports on whether perceived stress among female healthcare workers can reduce their turnover intention after proactive coping. In addition, the sequential model of future-oriented coping assumes that preventive coping will precede proactive coping. That is, in the early stage of coping with future stressful events, due to the relative remoteness of the stressful events and high uncertainty, individuals tend to adopt a defensive coping approach—preventive coping, i.e., to accumulate resources in advance. As time approaches, the future stressful event becomes clearer, individuals will take a more proactive and more purposeful response—proactive coping at this time [18]. Miao et al. found [15] that proactive coping methods have a more direct effect on behavioral outcomes, while preventive coping has an indirect effect on coping outcomes. Based on this, when individuals perceive stress, they may first adopt preventive coping and then act through proactive coping against detrimental outcomes. Therefore, we hypothesize that preventive coping and proactive coping may play a multiple mediation role in the relationship between female healthcare staff's perceived stress and turnover intention.

With the increase in women working and dual-income families, the structure of Chinese families and work-family relationships have changed dramatically. Previous foreign and domestic studies on women's work-family interface have mainly focused on work-family conflicts. For example, previous studies have reported that female healthcare workers have a significant conflict between work and family compared to males [19], which induces negative emotions, such as anxiety, depression [20], and job burnout [21]. A survey study showed that work-family imbalance is one of the main reasons for female workers to leave their jobs [22]. However, work-family conflict only partially describes the work-family relationship. The conflict and stress can be mitigated by psychosocial benefits originating in the work or family [23]. Therefore, work-family balance may be a more effective way to assess the work-family relationship among female healthcare workers. Work-family balance refers to individuals achieving goals and behaviors of role by negotiating and sharing with role partners in the work and family domains [24]. Role theory is an important theoretical basis for understanding and explaining work-family balance [25]. The expansion-enhancement theory is the core idea of role theory and holds that the multiple roles of individuals can not only conflict with each other but also promote each other [23]. An imbalance between work and family will lead to role conflicts, a depletion of individual resources, and an increase in the dual pressure of work and family, thereby inducing negative emotions. In contrast, a balance between work and family can reduce individual role conflicts, and the resources accumulated by individuals in the family can regulate work stress, relieve anxiety and tension, and improve well-being [26]. The most important two roles of healthcare staffs in work-family balance are their role as medical personnel and the role as a family member. For example, when both work-family roles are in good condition, medical personnel can be stimulated to obtain positive resources (such as emotional connection and social support) while engaging in family role activities, thereby improving family and work performance, achieving work-family dynamic balance, and thereby improve self-efficacy and work engagement. In addition, the stress response caused by role conflict can be alleviated through work-family balance [13], thereby effectively improving individual well-being and promoting physical and mental health [27]. Therefore, we hypothesize that work-family balance plays a mediating role between female healthcare staff's perceived stress and turnover intention.

**Fig. 1:**
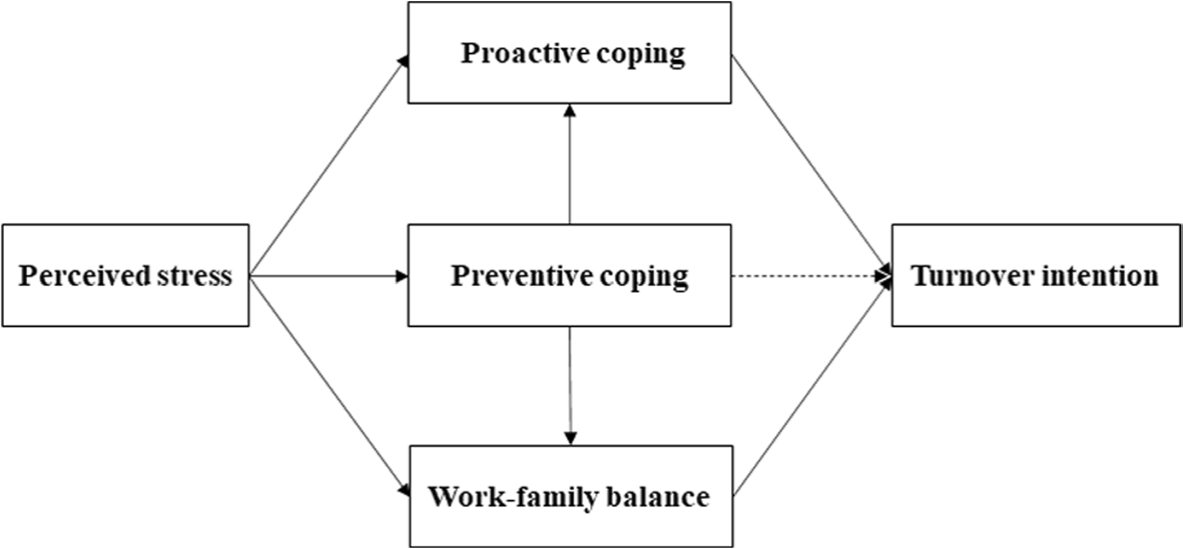
Conceptual model of this study

In conclusion, previous studies on the mediating effect of the relationship between perceived stress and turnover intention have mainly focused on burnout [28], work and family conflict [22], job satisfaction [29], and stress coping [30]. Few studies suggest that work-family balance and future orientation coping may play key roles in the relationship between perceived stress and turnover intention. Thus, this study is conducted to investigate the relationship between perceived stress and turnover intention among Chinese female healthcare workers, and to explore the mediating effect on this relationship from the perspectives of future orientation coping and work-family balance via a multiple-chain mediation model. In addition, we propose the following modeling hypotheses (Fig. 1):

- H1: Perceived stress positively predicts the turnover intention of female healthcare workers;
- H2: Preventive coping and proactive coping mediate the relationship between perceived stress and the turnover intention among female healthcare workers, and preventive coping predicts proactive coping (based on Lazarus' transactional model of stress and coping, as well as the sequential model of future-oriented coping);
- H3: Work-family balance mediates the relationship between perceived stress and turnover intention among female healthcare workers (based on the extension-enhancement theory);
- H4: Preventive coping, proactive coping, and work-family balance chain multiplicatively mediate the relationship between perceived stress and turnover intention among female healthcare workers (based on Lazarus' transactional model of stress and coping, the sequential model of future-oriented coping and the extension-enhancement theory).

## Methods

### Participants

This study was conducted using convenience sampling to recruit subjects of three hospitals in China. Inclusion criteria: (1) females; (2) healthcare professionals with a licensed practice; (3) informed consent and voluntary participation. Exclusion criteria: (1) males; (2) healthcare workers with a history of mental illness (e.g., depression, obsessive–compulsive disorder, et al.). G*Power was used to calculate the sample size and multiple linear regression was chosen. Setting an effect value of 0.15, a significance level of 0.05, a validity of 0.95, and 11 predictors resulted in a sample size of 178.

All subjects volunteered to participate in the study from February 28 to March 26, 2023 and completed the questionnaires anonymously by clicking on the questionnaire link (Questionnaire Star) with their digital device. To obtain informed consent, the first item of the online questionnaire was to inform the subject of the initiator of the survey, the content and purpose of the survey, the usage of the information, and then a question to ask whether he/she agreed to the survey. If agree, it will continue to show the subsequent questions items; if disagree, the survey will be stopped. A total of 495 questionnaires were completed and submitted successfully. To ensure invalid questionnaires, 59 cases were excluded that took less than 300 s. Because 20 volunteers were pre-tested in advance, and it took them (537.4 ± 228.48) seconds to fully understand and complete the questionnaires. In addition, 1 case was omitted due to a missing value for age, which was the covariate to be necessarily controlled. Finally, 435 valid questionnaires were obtained with a valid return rate of 87.88%.

### Measures

#### Perceived stress scale (PSS)

PSS is a 10-item scale (e.g., "During the past month, how much time did you feel out of control of important things in your life?") developed by Cohen et al. [31]. Five levels of scoring are used, with higher scores indicating greater perceived stress levels. In the present study, the α-coefficient was 0.879. The Bartlett's test of sphericity (*p* < 0.001), sample appropriateness test (KMO = 0.869), and confirmatory factor analysis (χ^2^/df = 4.418, CFI = 0.966, TLI = 0.940, RMSEA = 0.085, SRMR = 0.060) showed that the validity of the scale was acceptable.

#### Work-family balance scale (WFBS)

WFBS is a 17-item scale (e.g., "I will consciously regulate the allocation of time and energy between work and family") and scored on a 5-point scale, with higher scores indicating better work-family balance [32]. In this study, the α-coefficient was 0.811. The Bartlett's test of sphericity (*p* < 0.001), sample appropriateness test (KMO = 0.798) and confirmatory factor analysis (χ^2^/df = 3.586, CFI = 0.900, TLI = 0.869, RMSEA = 0.077, SRMR = 0.082) showed that the validity of the scale was acceptable.

### Future-oriented coping inventory (FOCI)

FOCI is a scale with 16 items and a 5-point, consists of two subscales: preventive coping (e.g., "I think beforehand to avoid dangerous situations") and proactive coping (e.g., "I sketch out my dreams and try to make them come true") [16, 33]. In the present study, the α-coefficient was 0.882 for the preventive coping subscale and 0.815 for the proactive coping subscale. The Bartlett's test of sphericity (*p* < 0.001), sample appropriateness test (KMO = 0.922) and confirmatory factor analysis (χ^2^/df = 3.943, CFI = 0.924, TLI = 0.907, RMSEA = 0.082, SRMR = 0.056) showed that the validity of the scale was acceptable.

#### Turnover intention scale (TIS)

TIS is a scale with 4 items (e.g., "I would rather do another job than my current job") and 5-point scale, with higher scores representing higher turnover intention [34]. In this study, the α-coefficient was 0.838. The Bartlett's test of sphericity (*p* < 0.001), sample appropriateness test (KMO = 0.922), and confirmatory factor analysis (χ^2^/df = 3.322, CFI = 0.994, TLI = 0.947, RMSEA = 0.073, SRMR = 0.018) showed that the validity of the scale was favorable.

### Statistical analysis

All data were analyzed using SPSS and Amos. Pearson correlation analysis was used to explore the relationship of the variables. Reliability and construct validity of the scale were assessed using Cronbach's alpha coefficient, Bartlett's test of sphericity, sample appropriateness test, and confirmatory factor analysis. The fit index (its acceptable value) of the scale including the α-coefficient (α > 0.8), the value of KMO (KMO > 0.5), Normed Chi-square (χ2/DF < 5), root mean square error of approximation (RMSEA < 0.08), standardized root mean square residual (SRMR < 0.08), Tucker-Lewis index (TLI > 0.90), and comparative fit index (CFI > 0.90) [35]. All statistical analyses were performed using *p* < 0.05 as the critical value for statistical significance. The bias-corrected bootstrap method based the PROCESS macro program was performed for path analysis. The number of bootstraps was set to 5000. If the 95% confidence interval of the bootstrap did not contain zero, the parameter estimate was significant; conversely, the parameter estimate was not significant [36].

## Results

### Common method variance

The Harman one-way test was used to test the common method bias. The results revealed that a total of 11 factors was generated without rotation, and the explanatory rate of the first factor was 27.03%, which was less than the critical criterion of 50% [37], indicating that there was no significant common method bias.

### Descriptive statistics and correlation analysis of variables

The mean age of the 435 validated subjects included was 33.59 years. 300 female medical workers were married (69.00%), 123 were unmarried (28.30%) and 12 were divorced (2.80%). 159 female subjects were childless (36.60%) and 275 had children (63.30%). Meanwhile, 282 had a bachelor's degree and below (64.80%), 79 had a master's degree (18.20%), and 74 had a doctoral degree (17.00%). In terms of post, 73 female subjects were doctors (16.80%), 314 were nurses (72.20%) and 48 were medical technicians (11.00%, Table 1).

**Table 1:** Descriptive statistics of participates

**Variables**	
**Age, *M* (*SD*)**	33.59(7.13)
**Marital status, n (%)**	
Married	300(69.00%)
Unmarried	123(28.30%)
Divorced	12(2.80%)
**Children, n (%)**	
No	159(36.60%)
Yes	275(63.20%)
**Education, n (%)**	
Bachelor's degree and below	282 (64.80%)
Master's degree	79 (18.20%)
Doctoral degree	74(17.00%)
**Post, n (%)**	
Doctor	73 (16.80%)
Nurse	314 (72.20%)
Medical technician	48 (11.00%)

Correlation analysis was shown in Table 2. In terms of the relationship between demographic variables and predictor variables, only age and education were significantly associated with perceived stress (*r*_age_ = -0.132, *p* = 0.006), preventive coping (*r*_age_ = 0.161, *p* = 0.001), proactive coping (*r*_age_ = 0.123, *p* = 0.010), and turnover intention (*r*_age_ = -0.127, *p* = 0.008; *r*_education_ = -0.153, *p* = 0.001) in the female staffs. In terms of the relationship among predictor variables, perceived stress was negatively correlated with preventive coping (*r* = -0.463, *p* < 0.001), proactive coping (*r* = -0.531, *p* < 0.001), and work-family balance (*r* = -0.587, *p* < 0.001), respectively. Meanwhile, perceived stress was positively correlated with turnover intention (*r* = 0.507, *p* < 0.001). The turnover intention was negatively correlated with preventive coping (*r* = -0.303, *p* < 0.001), proactive coping (*r* = -0.271, *p* < 0.001) and work-family balance (*r* = -0.571, *p* < 0.001), respectively. Proactive coping, preventive coping and work-family balance (*r*_preventive coping_ = 0.736, *r*_preventive coping_ = 0.448, *r*_proactive coping_ = 0.471, *p*_*s*_ < 0.001) were positively correlated with each other.

**Table 2:** correlations among variables

Variables	1	2	3	4	5	6	7	8	9	10
1 Marital status	1									
2 Children	-0.648^***^	1								
3 Post	-0.023	-0.006	1							
4 Education	-0.054	0.030	-0.118^*^	1						
5 Age	-0.413^***^	0.581^***^	0.001	0.099^*^	1					
6 Perceived stress	0.012	-0.032	-0.074	− 0.038	− 0.132^**^	1	1			
7 Work-family balance	0.008	-0.017	0.076	− 0.048	0.020	-0.587^***^	1			
8 Turnover intention	0.047	-0.093	0.023	− 0.127^**^	− 0.153^**^	0.507^***^	-0.571^**^	1		
9 Preventive coping	0.057	-0.077	-0.059	0.161^**^	0.010	-0.463^***^	0.448^***^	− 0.303^***^	1	
10 Proactive coping	0.011	-0.005	-0.034	0.123^**^	0.077	-0.531^***^	0.471^***^	− 0.271^***^	0.736^***^	1

^*^indicates significant at the 0.05 level, i.e., *p* < 0.05

^**^indicates significant at the 0.01 level, i.e., *p* < 0.01

^***^indicates significant at the 0.001 level, i.e., *p* < 0.001

### Chain mediating effect test

The chain mediating effect test was conducted using Model 6 in the SPSS PROCESS macro program [36] based on the results of the correlation analysis, with perceived stress as a predictor variable, turnover intention as an outcome variable, preventive coping, proactive coping and work-family balance as mediating variables with controlling for education and age. The regression analysis is shown in Table 3. Perceived stress positively predicted turnover intention (*β* = 0.152, *p* < 0.001) and negatively predicted preventive coping (*β* = -0.366, *p* < 0.001), proactive coping (*β* = -0.171, *p* < 0.001), and work-family balance (*β* = -0.663, *p* < 0.001); Preventive coping positively predicted proactive coping (*β* = 0.579, *p* < 0.001) and work-family balance (*β* = 0.301, *p* = 0.004); Proactive coping positively predicted work-family balance (*β* = 0.256, *p* = 0.029) and turnover intention (*β* = 0.129, *p* = 0.004); Work-family balance negatively predicted turnover intention (*β* = -0.176, *p* < 0.001).

**Table 3:** Regression analysis of variables in the chained multiple mediation model

Outcome variables	Predictor variables	*β*	*SE*	*P*	95%CI	*R*^*2*^	*F*
Preventive coping	Education	0.859	0.242	< 0.001	[0.383, 1.335]	0.240	45.199^***^
	Age	− 0.044	0.026	0.098	[− 0.096, 0.008]		
	Perceived stress	− 0.366	0.033	< 0.001	[− 0.431, − 0.300]		
Proactive coping	Education	0.054	0.167	0.746	[− 0.275, 0.384]	0.589	153.606^***^
	Age	0.022	0.018	0.219	[− 0.013, 0.058]		
	Perceived stress	− 0.171	0.026	< 0.001	[− 0.221, − 0.120]		
	preventive coping	0.579	0.033	< 0.001	[0.514, 0.643]		
Work-family balance	Education	− 1.071	0.406	< 0.001	[− 1.869, − 0.274]	0.405	58.213^***^
	Age	− 0.043	0.044	0.330	[− 0.129, 0.043]		
	Perceived stress	− 0.663	0.065	< 0.001	[− 0.792, − 0.535]		
	preventive coping	0.301	0.105	0.004	[0.096, 0.506]		
	proactive coping	0.256	0.117	0.029	[0.026, 0.486]		
turnover intention	Education	− 0.560	0.155	< 0.001	[− 0.865, − 0.255]	0.412	49.869^***^
	Age	− 0.047	0.017	0.005	[− 0.080, − 0.015]		
	Perceived stress	0.152	0.028	< 0.001	[0.097, 0.206]		
	Preventive coping	− 0.051	0.040	0.204	[− 130, 0.028]		
	Proactive coping	0.129	0.045	0.004	[0.041, 0.217]		
	Work-family balance	− 0.176	0.018	< 0.001	[− 0.212, − 0.140]		

^***^indicates significant at the 0.001 level, i.e., *p* < 0.001

**Fig. 2:**
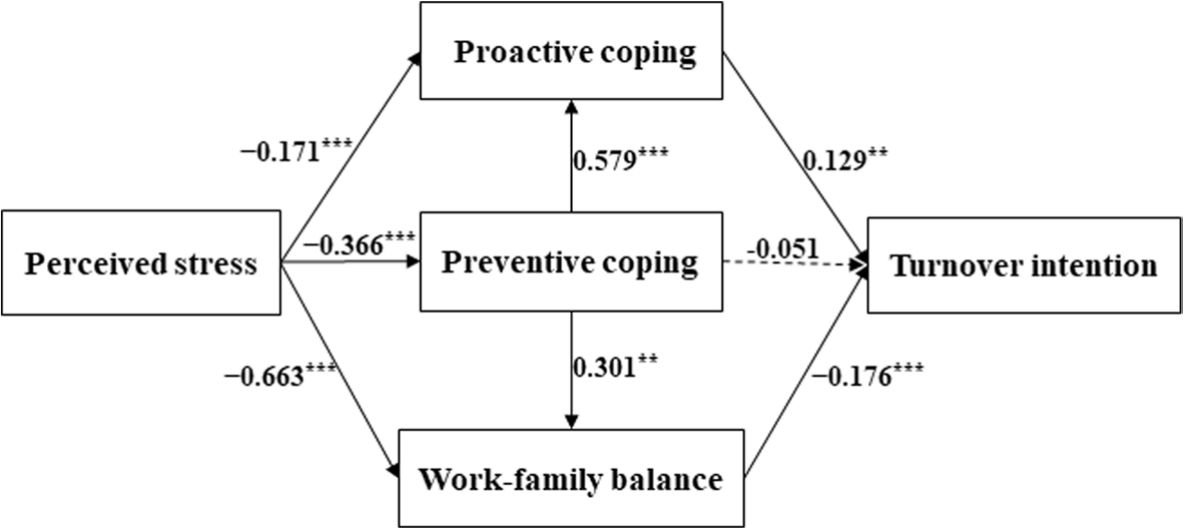
A serial multiple mediation model for preventive coping, proactive coping, and work-family balance

The mediation effect analysis (Table 4) showed that the mediation effects of preventive coping, proactive coping, and work-family balance in perceived stress and turnover intention are significant. In addition, the chain multiple mediation effects of preventive coping, proactive coping, and work-family balance in perceived stress and turnover intention were also significant. The total indirect effect value was 0.123 and the effect size was 0.447. The chain multiple mediation mechanism of preventive coping, proactive coping and work-family balance includes seven paths: path 1: Perceived stress → Preventive coping → Turnover intention; path 2: Perceived stress → Proactive coping → Turnover intention; path 3: Perceived stress → Work-family balance → Turnover intention; path 4: Perceived stress → Preventive coping → Proactive coping → Turnover intention; path 5: Perceived stress → Preventive coping → Work-family balance → Turnover intention; Path 6: Perceived stress → Preventive coping → Work-family balance → Turnover intention; Path 7: Perceived stress → Preventive coping → Proactive coping → Work-family balance → Turnover intention. Among the 7 paths, 2, 3, 4 and 5 path were significant with an effect size of -0.080, 0.425, -0.098 and 0.069, respectively. Path 1, 6 and 7 were not significant (Fig. 2).

**Table 4:** Bootstrap analysis of the mediating effects test

	Indirect effect value	Boot SE	Boot LLCI	Boot UlCI	Effect size
Total indirect effects	0.123	0.022	0.081	0.168	0.447
Perceived stress→Preventive coping→ Turnover intention	0.018	0.018	− 0.017	0.055	
Perceived stress→Proactive coping→ Turnover intention	− 0.022	0.010	− 0.042	− 0.003	** − 0.080**
Perceived stress→Work-family balance→ Turnover intention	0.117	0.018	0.083	0.156	**0.425**
Perceived stress→Preventive coping→Proactive coping→ Turnover intention	− 0.027	0.012	− 0.052	− 0.004	** − 0.098**
Perceived stress→Preventive coping→Work-family balance→ Turnover intention	0.019	0.009	0.003	0.039	**0.069**
Perceived stress→Proactive coping→Work-family balance→ Turnover intention	0.008	0.005	− 0.001	0.017	
Perceived stress→Preventive coping→Proactive coping→Work-family balance→ Turnover intention	0.010	0.006	− 0.011	0.090

## Discussion

This study explored the influence and mechanism of perceived stress on turnover intention in female healthcare staffs through the analysis of the mediating effect under the control of the age and education. The results demonstrated that perceived stress can not only directly and positively predict the level of turnover intention in female healthcare staffs, but also indirectly affect turnover intention through proactive coping or work-family balance. Also, perceived stress can indirectly affect the turnover intention in female healthcare staffs through multiple mediations of preventive coping → proactive coping or work-family balance.

Firstly, this study found that the level of perceived stress is generally high in female healthcare staffs, and the perceived stress can directly and positively predict the turnover intention, that is, as the perceived stress level increases in female healthcare staffs, their turnover intention will also increase. This finding is consistent to a previous report that the greater the perceived work pressure, the more likely the healthcare staffs are to leave their current jobs [32, 33]. According to the theory of psychological stress, female roles, occupational exposure, and high-intensity work could be threatening stressors, which can induce negative emotions such as tension and anxiety in individuals, thereby affect their work performance and turnover intention [38]. Since the outbreak of the COVID-19 epidemic, the work intensity and difficulty as well as the risk of occupational exposure have increased significantly in healthcare staffs, especially, in female healthcare staffs, which result in a decline in work efficiency [38] and an increase in individual’s stress level. At the same time, the decline in work efficiency will further lead to an increase in stress levels [20], which will eventually increase the tendency to leave the job.

Secondly, this study found that the perceived stress can negatively predict the proactive coping and preventive coping. This indicates that female healthcare workers are less likely to choose prevention and proactive coping when they perceive stress. This may be because stress induces burnout and a sense of helplessness in female healthcare workers [39], which makes them passively cope and less likely to take positively coping, such as preventive and proactive coping. Meanwhile, proactive coping plays a mediating role between perceived stress and turnover intention. This means that perceived stress not only has a direct effect on turnover intention in female healthcare staffs, but also indirectly affects turnover intention through proactive coping. This is to say that female healthcare workers with high stress levels are less willing to take proactive coping, such as self-improvement and growth, which can further increase their turnover intention. Chronic work-related stress is one of the causes for the female healthcare staff's unhealthy behaviors [40, 41], but adaptive coping styles can effectively reduce stress intensity, psychological and behavioral burnout [42]. Therefore, this study confirms that as an adaptive coping method, proactive coping plays a protective role in the process of perceived stress and effectively predicts the turnover intention in female healthcare staffs and alleviates the promoting role of high perceived stress in turnover intention. Those implicate that the managers of female healthcare staffs should pay more attention to improving their adaptive coping style through training or lectures, and actively coping with the pressure at work, subsequently improving their work efficiency, and increasing self-efficacy and work enthusiasm, thereby reducing their turnover tendency.

Thirdly, this study demonstrated that work-family balance plays a mediating role between perceived stress and turnover intention in female healthcare staffs. This means if female healthcare staffs can identify and balance the two roles of work and family when feel pressure, they can reduce their turnover intention, and vice versa. Based on the resource compensation effect, the resources accumulated by individuals in the family can adjust their work pressure and improve their work enthusiasm. Yang et al. found that recognition of family roles can promote individual’s work engagement [43]. Collectively, this study suggests that under a work-family balance, female healthcare staffs can establish intimate connections with family members through family activities, while the emotional connection and family support can not only effectively regulate the level of stress, but also relieve the physiological reactions and uncomfortable behaviors triggered by stress, can improve self-efficacy and career accomplishment, and effectively reduce the intention to leave [44]. In addition, it has demonstrated that during the COVID-19 pandemic, due to the increase in the absolute demand for medical nursing knowledge at home and the significant contribution of healthcare staffs to society [40, 45], female healthcare staffs got more supports from family, which improved their self-efficacy, thereby reduced their turnover intention.

This study also found that preventive coping, proactive coping, and work-family balance play multiple mediation roles between perceived stress and turnover intention in female healthcare workers. Specifically, perceived stress not only directly affects the turnover intention in female healthcare workers through proactive coping, but also effectively predicts the effect of proactive coping on turnover intention through preventive coping, which is consistent with the sequence model of future-oriented coping [18]. In addition, preventive coping can also mediate the relationship between perceived stress and turnover intention in female healthcare staffs through work-family balance. When healthcare staffs perceive stress, they will assess potential stressors and accumulate resources, and then pre-respond based on prevention, and finally reduce their turnover intention. According to expansion-enhancement theory, healthcare staffs can obtain resources from the performance of multiple roles to overcome the role pressure. That is to say that in the prevention and response stage, the positive emotions generated by healthcare staffs and family members in family activities can effectively promote the role activities [44]. While achieving self-improvement, they can also fully mobilize resources (such as supportive resources) through proactive coping to alleviate the negative emotions of healthcare staffs, improve their behavioral efficiency, enhance their work engagement, and reduce their turnover intention. This not only further supports the sequential relationship between preventive coping and proactive coping [18], that is, the two coping methods are not completely independent, but complement each other in the process of coping with potential stress, and provide phased intervention ideas for healthcare staffs to deal with potential stressors. This indicated that it is necessary to specifically analyze the stress response stages (preventive coping and proactive coping) and carry out targeted phased interventions in the process of dealing with potential stressors. At the same time, in order to better cope with future stressors, the important role of work-family balance should also be fully played, so as to better cope with challenges, maintain the physical and mental health of female healthcare staffs, and improve their work efficiency and satisfaction.

We acknowledge that this study has the following limitations: (1) During the questionnaire survey, the COVID-19 epidemic has not been fully released, which may be a potential unpredictable stressor with certain specificity, but this study did not further refine the types of potential stressors. Therefore, future research can further distinguish the specific coping styles for differently potential stressors; (2) This study built a chain model only based on horizontal data, and the results verified the sequence model of future-oriented coping in the female healthcare staffs, but the longitudinal data are limited. Therefore, future studies can conduct longitudinal mediation and cross-lag analysis based on longitudinal data to fully verify the sequence model of future orientation coping; (3) In this study, only female subjects were studied, and future studies can enrich male medical subjects and explore gender differences in coping with potential stressors and specific effects on coping outcomes.

## Conclusions

This study found that perceived stress affects the level of turnover intention in female healthcare workers through preventive coping, proactive coping, and work-family balance. In addition, the sequential model of future-oriented coping is validated in female healthcare staffs, which means that it is necessary to specifically analyze the stress response stages (preventive coping and proactive coping) and carry out targeted phased interventions in the process of dealing with potential stressors. In order to better cope with future stressors, the important role of work-family balance should be fully played, so as to better cope with challenges, maintain the physical and mental health of female healthcare workers, and improve their work efficiency and satisfaction. Moreover, serial multiple mediation analysis can help establish and verify the mediation model and further develop and expand the theories.

## Data Availability

No datasets were generated or analysed during the current study.

## References

[1] Baptista S, Teixeira A, Castro L, Cunha M, Serrão C, Rodrigues A (2021). Physician burnout in primary care during the COVID-19 Pandemic: a cross-sectional study in portugal. J Prim Care Community Health.

[2] Tao SY, Liu W, Hao YH, Song WJ, Xue YX, Wang JH (2020). Analysis of the current situation of clinicians' willingness to leave in four tertiary hospitals in western Heilongjiang Province. Chinese Hospitals.

[3] Wang Y, Yuan H (2018). What is behind high turnover: a questionnaire survey of hospital nursing care workers in shanghai, China. BMC Health Serv Res.

[4] Peltokorpi V, Allen DG, Froese F (2015). Organizational embeddedness, turnover intentions, and voluntary turnover: the moderating effects of employee demographic characteristics and value orientations. J Organ Behav.

[5] Huang TL, Wong MK, Shyu YIL, Ho LH, Yeh JR, Teng CI (2021). Reducing turnover intention to improve care outcome: a two-wave study. J Adv Nurs.

[6] Holland P, Tham TL, Sheehan C, Cooper B (2019). The impact of perceived workload on nurse satisfaction with work-life balance and intention to leave the occupation. Appl Nurs Res ANR.

[7] Grant S, Davidson J, Manges K, Dermenchyan A, Wilson E, Dowdell E (2020). Creating healthful work environments to deliver on the quadruple aim: a call to action. J Nurs Adm.

[8] Morens DM, Daszak P, Markel H, Taubenberger JK (2020). Pandemic COVID-19 joins history’s pandemic legion. mBio.

[9] Nazir T, Umer M, Najam M, Nawab S, Maqsoom A, Shafi K (2022). Impact role stress on turnover intentions of pakistan’s healthcare workers: Mediating and moderating role of organizational cynicism and self-efficacy. PLoS ONE.

[10] Bohlken J, Schömig F, Lemke MR, Pumberger M, Riedel-Heller SG (2020). COVID-19 pandemic: stress experience of healthcare workers - a short current review. Psychiatr Prax.

[11] Heponiemi T, Presseau J, Elovainio M (2016). On-call work and physicians’ turnover intention: the moderating effect of job strain. Psychol Health Med.

[12] Folkman S, Lazarus RS, Gruen RJ, Delongis A (1986). Appraisal, coping, health status, and psychological symptoms. J Pers Soc Psychol.

[13] Xiong SC, Xu Y, Zhang B, Zhu LF, Su ZF (2021). The relationship between acute stress and nurses' work withdrawal under major public health events: a mediated model with moderation. Chin J Clin Psychol.

[14] Schwarzer R. Tenacious Goal Pursuits and Striving Toward Personal Growth: Proactive Coping. 2002; Available from: http://doc.paperpass.com/foreign/rgArti2002141584705.html. Cited 2023 May 6.

[15] Miao M, Wang YQ, Ye Q, Ke Q, Gan YQ (2017). alidation of a sequential model of future-oriented coping in prospective newlywed individuals. Chinese J Clin Psychol.

[16] Gan Y, Yang M, Yan Z, Zhang Y (2007). The two-factor structure of future-oriented coping and its mediating role in student engagement. Personal Individ Differ.

[17] Hu H, Wang C, Lan Y, Wu X (2022). Nurses’ turnover intention, hope and career identity: the mediating role of job satisfaction. BMC Nurs.

[18] Gan YQ (2011). A two-stage sequential model of future-oriented coping and its time-perspective mechanism. Adv Psycholog Sci.

[19] Wu Y-F, Wang P-C, Chen Y-C (2018). Gender differences and work-family conflicts among emergency physicians with intention to leave. Emerg Med Int.

[20] Pappa S, Ntella V, Giannakas T, Giannakoulis VG, Papoutsi E, Katsaounou P (2020). Prevalence of depression, anxiety, and insomnia among healthcare workers during the COVID-19 pandemic: a systematic review and meta-analysis. Brain Behav Immun.

[21] Gold KJ, Kuznia AL, Laurie AR, Williams CB (2021). Gender differences in stress and burnout: department survey of academic family physicians. J Gen Intern Med.

[22] José Aurelio Medina-Garrido, José María Biedma-Ferrer, María Vanessa Rodríguez-Cornejo. I Quit! Effects of Work-Family Policies on the Turnover Intention. Int. J. Environ. Res. Public. Health. 2021;(4). Available from: http://pubmed.ncbi.nlm.nih.gov/33669281/. Cited 2023 May 6. 10.3390/ijerph18041893 PMC7920080 33669281

[23] Macdermid M (1996). Multiple roles and the self: a theory of role balance. J Marriage Fam.

[24] Grzywacz JG, Carlson DS (2007). conceptualizing work—family balance: implications for practice and research. Adv Dev Hum Resour SAGE Pub.

[25] Peltokorpi V, Cieply I, Froese FJ. Woman’s work: The moderating effects of gender role orientations between the relationships of work-family conflict with voluntary turnover and being valued by one’s spouse. Int J. Psychol. 2023 Dec 1. 10.1002/ijop.13095 38041419

[26] Grandey AA, Cropanzano R (1999). The conservation of resources model applied to work-family conflict and strain. J Vocat Behav.

[27] Barnett MD, Martin KJ, Garza CJ. Satisfaction With Work–Family Balance Mediates the Relationship Between Workplace Social Support and Depression Among Hospice Nurses. J. Nurs. Scholarsh. 2019;51. Available from: http://pubmed.ncbi.nlm.nih.gov/30570211/. Cited 2023 May 6. 10.1111/jnu.12451 30570211

[28] Phillips C (2020). Relationships between workload perception, burnout, and intent to leave among medical-surgical nurses. Int J Evid Based Healthc.

[29] Labrague LJ, McEnroe-Petitte DM, Gloe D, Tsaras K, Arteche DL, Maldia F (2017). Organizational politics, nurses’ stress, burnout levels, turnover intention and job satisfaction. Int Nurs Rev.

[30] Yang T, Jin X, Shi H, Liu Y, Guo Y, Gao Y (2021). Occupational stress, distributive justice and turnover intention among public hospital nurses in China: a cross-sectional study. Appl Nurs Res ANR.

[31] Cohen S. Perceived stress in a probability sample of the United States. Soc. Psychol. Health . Thousand Oaks, CA, US: Sage Publications, Inc; 1988. p. 31–67.

[32] Lin XY, Wang YL, Hao YJ, Li HJ (2016). A study on the mechanism of the influence of leader-member exchange on work-family balance: The mediating role of work flexibility ability and the moderating role of work flexibility intention. Manage Rev.

[33] Greenglass E, Schwarzer R, Jakubiec D, Fiksenbaum L, Taubert S. The Proactive Coping Inventory (PCI): A Theory-Guided Multidimensional Instrument. 1999; Available from: http://www.nuokui.com/doc/sBp35MG2crjI.html. Cited 2023 May 6.

[34] Scott CR, Connaughton SL, Diaz-Saenz HR, Maguire K, Ramirez R, Richardson B (1999). The Impacts of communication and multiple identifications on intent to leave: a multimethodological exploration. Manag Commun Q..

[35] Zhonglin W, Kit-Tai H, Marsh HW (2004). Structural equation model testing: cutoff criteria for goodness of fit indices and chi-square test. Acta Psychol Sin.

[36] Igartua JJ, Hayes AF (2021). Mediation, moderation, and conditional process analysis: concepts, computations, and some common confusions. Span J Psychol.

[37] Malhotra NK, Kim SS, Patil A. Common Method Variance in IS Research: A Comparison of Alternative Approaches and a Reanalysis of Past Research. Manag. Ence. 2006;52. Available from: http://econpapers.repec.org/article/inmormnsc/v_3a52_3ay_3a2006_3ai_3a12_3ap_3a1865-1883.htm. Cited 2023 May 6.

[38] Labrague LJ, de Los Santos JAA (2021). Fear of COVID-19, psychological distress, work satisfaction and turnover intention among frontline nurses. J Nurs Manag.

[39] Ramírez-Elvira S, Romero-Béjar JL, Suleiman-Martos N, Gómez-Urquiza JL, Monsalve-Reyes C, Cañadas-De la fuente GA (2021). Prevalence, risk factors and burnout levels in intensive care unit nurses: a systematic review and meta-analysis. Int J Environ Res Public Health.

[40] Schutz V, Shattell M. Impact of COVID-19: What Does It Mean For Nurses and Health Systems? J. Psychosoc. Nurs. Ment. Health Serv. 2020;(8). Available from: http://pubmed.ncbi.nlm.nih.gov/32744640/. Cited 2023 May 6. 10.3928/02793695-20200707-01 32744640

[41] Caperelli Gergel MC, Terry DL (2022). Giving 200%: workplace flexibility and provider distress among female physicians. J Healthc Leadersh.

[42] Biaczyk K, Wyszkowska Z, Maciej Bieliński. Affective Temperament is Associated with Stress Coping Strategies and Work Stress Perception Among Polish Bank Employees. Psychol. Res. Behav. Manag. 2020; Available from: http://pubmed.ncbi.nlm.nih.gov/33414647/. Cited 2023 May 6. 10.2147/PRBM.S280156 PMC7783194 33414647

[43] Yang BY, Su SQ (2022). The relationship between family role identity and work engagement: a mediated model with moderation. Chin J Clin Psychol.

[44] Tselebis A, Lekka D, Sikaras C, Tsomaka E, Pachi A (2020). Insomnia, perceived stress, and family support among nursing staff during the pandemic crisis. Healthcare.

[45] Green-Laughlin DL (2020). COVID-19: a closer lens. Issues Ment Health Nurs.

